# A high-caloric diet rich in soy oil alleviates oxidative damage of skeletal muscles induced by dexamethasone in chickens

**DOI:** 10.1080/13510002.2017.1405494

**Published:** 2017-11-20

**Authors:** Hongchao Jiao, Kaifeng Zhou, Jingpeng Zhao, Xiaojuan Wang, Hai Lin

**Affiliations:** a Shandong Provincial Key Laboratory of Animal Biotechnology and Disease Control and Prevention, Shandong Agricultural University, Taian, Shandong, People's Republic of China; b Shandong Extension Station of Animal Husbandry, Jinan, Shandong, People's Republic of China

**Keywords:** Glucocorticoid, oxidative damage, muscle, soy oil, mitochondria, stress

## Abstract

**Objective:** Glucocorticoids (GCs) can induce oxidative damage in skeletal muscles. The purpose of this study was to demonstrate a high caloric (HC) diet rich in soy oil would change the oxidative stress induced by a GC.

**Methods:** The effect of dexamethasone (DEX) and HC diet on oxidative stress in plasma, skeletal muscles (*M. pectoralis major*, PM; *M. biceps femoris*, BF), and mitochondria were determined. The biomarkers of oxidative damage and antioxidative enzyme activity were determined. The fatty acid profile of muscles and the activities of complex I and II in mitochondria were measured.

**Results:** The results showed that DEX increased the concentrations of oxidative damage markers in plasma, muscles, and mitochondria. The activity of complex I was significantly suppressed by DEX. DEX-chickens had higher proportions of polyunsaturated fatty acids and lower proportions of monounsaturated fatty acids in the PM. A HC diet decreased the levels of oxidative damage biomarkers in plasma, muscles, and mitochondria. The interaction between DEX and diet suppressed the activities of complex I and II in HC-chickens.

**Discussion:** Oxidative damage in skeletal muscles and mitochondria was the result of GC-induced suppression of the activity of mitochondrial complex I. A HC diet improved the antioxidative capacity and reduced the oxidative damage induced by the GC.

## Introduction

An imbalance between the production of reactive oxygen species (ROS) and the capacity of antioxidant systems results in oxidative damage and disease [1,2]. Oxidative stress in skeletal muscles is relevant to the development of insulin resistance [3,4]. During stress, an increase in circulating levels of glucocorticoids (GCs), the final effectors of the hypothalamic–pituitary–adrenal axis, is involved in stress-induced oxidative damage in poultry [5–8] and mammals [9–11]. Mitochondria are the primary sites of ROS production [12–14]. During heat stress, impaired mitochondrial function in skeletal muscles induces oxidative damage in the skeletal muscles of broilers [15,16]. However, the influence of GCs on mitochondrial function in skeletal muscles and the resulting effects on the redox balance remain to be elucidated.

Dietary caloric intake is linked to antioxidant capacity. Under physiological conditions, the level of caloric intake is an important factor in modulating the total antioxidant capacity in the plasma [17,18]. The antioxidant defenses in skeletal muscle mitochondria increase in rats maintained for 2 weeks under caloric restriction [19]. Dietary fat levels can influence the antioxidant capacity of cells and tissues either positively or negatively. When fed an isocaloric, high-fat diet, the levels of antioxidant defenses increase in rat skeletal muscle [20]. However, the reduction in oxidative stress associated with a low caloric intake is lost when the proportion of dietary fat increases [21,22]. By contrast, moderate levels of dietary fat in the form of plant oils rich in polyunsaturated fatty acids can benefit the redox balance [23,24]. Hence, we hypothesized that GC-induced oxidative stress in skeletal muscle would be affected by a high-caloric (HC) diet rich in soy oil. The objective of this study was to explore the interaction between a GC and a HC diet on oxidative stress in skeletal muscle tissue of chickens.

## Materials and methods

### Ethical statement

The study was approved by the Animal Care and Use Committee of Shandong Agricultural University and was performed in accordance with the ‘Guidelines for the Use of Experimental Animal’ of the Ministry of Science and Technology (Beijing, People’s Republic of China).

### Animals and experimental design

Day-old male chicks (Arbor Acres, 40.0 g) were obtained from a local hatchery (Dabao Breeding Co., Taian, Shandong, People’s Republic of China), and all chicks were reared in an environmentally controlled room. The brooding temperature was 35°C (65% relative humidity, RH) for the first 2 days and then was gradually reduced to 24°C (45% RH) by day 21. All birds had free access to food and water during the rearing and experimental periods. Two randomized controlled trials were conducted.

#### Experiment 1

At 14 days of age, 24 broilers with body weight approximating the mean body weight were selected and randomly divided into two groups of 12 chickens each. At 16 days of age, one group of chickens received daily subcutaneous injections of dexamethasone (DEX, 2.0 mg/kg body mass; [8]) for 3 days, and the other group of chickens was sham-treated with the same volume of saline (Control). All chickens received a commercial starter diet with 21.0% crude protein and 12.37 MJ/kg of metabolizable energy (ME).

At 24 h after the last injection, eight chickens were randomly selected from each group. A blood sample was drawn from a wing vein with a heparinized syringe within 30 s and collected in a tube placed on ice. Plasma was obtained after centrifugation at 400 ×*g* for 10 min at 4°C and was stored at −20°C for further analysis. Thereafter, the chickens were killed via exsanguination following cervical dislocation. Muscle samples were obtained from the left *M. pectoralis major* (PM, fast-twitch glycolytic fibers) and the left *M. biceps femoris* (BF, slow-twitch oxidative fibers) and were cut in half. The proximal halves were used for mitochondria isolation, and the distal halves were snap-frozen in liquid nitrogen and stored at −80°C for further analysis.

#### Experiment 2

At 1 day of age, 150 broilers were randomly divided into two groups of 75 chicks each and fed with either a normal diet (ND, 12.4 MJ ME/kg, 3.0% soybean oil, and 20.0% crude protein) or a HC diet (15.1 MJ ME/kg, 12.8% soybean oil, and 20.0% crude protein). At 16 days of age, 24 chickens were randomly selected from each dietary group, divided into two subgroups of 12 chickens each, and then subjected to one of two treatments: daily subcutaneous injections of DEX (2.0 mg/kg body mass) or sham injections with saline (Control). After 3 days of injections, eight chickens were randomly selected from each treatment and sampled for blood and skeletal muscle as described in experiment 1.

### Plasma variable measurements

The levels of plasma lipid peroxidation were determined via the spectrophotometric measurement of thiobarbituric acid reacting substances, expressed as nmol of malondialdehyde (MDA) [6,7]. The concentrations of urate and 8-hydroxydeoxyguanosine (8-OHdG) and the activities of catalase (CAT), total superoxide dismutase (T-SOD), and glutathione peroxidase (GSH-Px) were measured using commercial diagnostic kits (Jiancheng Bioengineering Institute, Nanjing, Jiangsu, People’s Republic of China). All kits used in the present study have been successfully used in previous poultry studies [8,25]. Allantoin levels were measured via high-performance liquid chromatography (SPD-10AVP; Shimadzu, Kyoto, Japan) using a modification of the method of Grootveld and Halliwell [26], as described by Huang et al. [15].

### Oxidative damage biomarkers and enzyme activities in muscle tissue

Muscle tissue samples were homogenized in 9 volumes of 10 mM sodium phosphate buffer (pH 7.4) containing 1.15% potassium chloride. Following centrifugation of the homogenates (400×*g* for 10 min at 4°C), the supernatant was used for further measurements. The concentrations of thiobarbituric acid reacting substances in skeletal muscle tissues were measured as described by Lin et al. [6,7] and are expressed as nmol of MDA/mg protein. The protein carbonyl content and the CAT, T-SOD, and GSH-Px activities were measured using commercial kits as described above. The concentration of 8-OHdG, an oxidized nucleoside of DNA that is frequently measured as an indicator of DNA damage [27], was determined using a commercial kit (R & D systems Comp., No. 201005).

### Muscle fatty acid composition determination

Fatty acid composition was measured according to Gao et al. [8]. Briefly, total lipid extracts of muscle samples were transmethylated into fatty acid methyl esters and separated via gas chromatography (GC2014B; Shimadzu, Kyoto, Japan). Aliquots of 1 μl were injected into a DB-23 capillary column (30 m long × 0.25 mm i.d., 0.25 μm thick; Agilent J & W Technologies, USA). Nitrogen was used as the carrier gas at a constant flow rate of 24 ml/min. The following oven temperature program was used: 150°C held for 2 min, increased to 180°C at 6°C/min and held for 2 min, and then increased to 210°C at 5°C/min and held at 210°C for 2 min. Peaks were separated using a flame ionization detector and were quantified and identified with electric integrator (CR-8A; Shimadzu, Kyoto, Japan)-based pure standard mixtures (Sigma, St. Louis, MO, USA). The contents of saturated fatty acids (SFAs), monounsaturated fatty acids (MUFAs) and polyunsaturated fatty acids (PUFAs) were measured, and the ratios of MUFAs to SFAs and PUFAs to SFAs were calculated.

### Oxidative damage and antioxidative enzyme activity in mitochondria

Muscle cell mitochondria were isolated according to Bhattacharya et al. [28] and Bottje et al. [29], with some modifications. All media were ice-cold, and procedures were performed on ice or at 4°C to avoid the possible influence of artifacts. Muscle samples were cleaned of connective tissue and fat, and finely minced in an empty dish. Minced tissues were incubated in 10 ml of medium A (100 mM sucrose, 10 mM EDTA, 100 mM Tris-HCl, and 46 mM KCl [pH 7.4]) containing 0.02% nagarase for 5 min on ice. The tissues were then washed and resuspended in 10 ml of medium A plus 0.5% BSA. The minced tissues were then homogenized. The homogenate was centrifuged (500×g for 10 min) at 4°C to separate the suspension, and the resulting supernatant was centrifuged (12,000×*g* for 10 min) again to obtain the mitochondrial pellet, which was resuspended and washed in 10 ml of isolation medium A plus 0.5% BSA. Mitochondria were then pelleted via centrifugation (12,000×g for 10 min) in the incubation medium (230 mM mannitol, 70 mM sucrose, 20 mM Tris-HCl, and 5 mM KH_2_PO_4_ [pH 7.4]). The resulting pellet was resuspended in 2 ml of the incubation medium and placed on ice for later use in the functional studies. The activities of the respiratory chain complexes were assessed with an ultraviolet spectrophotometer using the method described by Bottje et al. [29] and Iqbal et al. [30]. The respiratory chain complex activities are expressed in units of activity per minute per milligram of mitochondrial protein.

Protein carbonyl contents, the ability to inhibit hydroxyl radicals (AIHR), and the mitochondrial activities of CAT, T-SOD, and GSH-Px and content of 8-OHdG were all determined using commercial kits as described above.

### Statistical analyses

All data from experiment 1 were subjected to a one-way ANOVA to test the main effect of the DEX treatment. In experiment 2, a two-way ANOVA was used to analyze the main effects of DEX and diet and their interaction. Differences between means were further assessed using Duncan's multiple range analysis. *P* < .05 was considered statistically significant.

## Results

### Oxidative damage biomarkers and enzyme activities in plasma and muscle tissue in experiment 1

**Figure 1.**
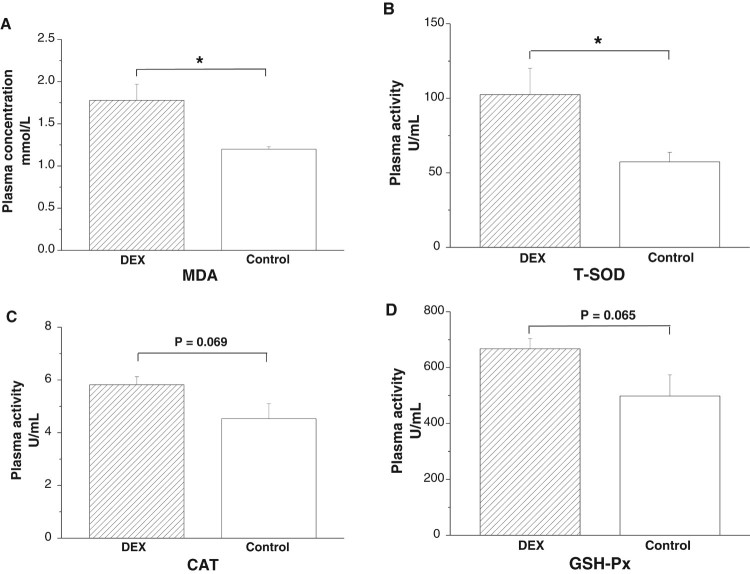
Effects of DEX (2.0 mg/kg body weight) on plasma parameters of the broilers in experiment 1. Data are expressed as the mean ± SEM (*n* = 8). *P < .05. (A) MDA; (B) T-SOD; (C) CAT; (D) GSH-Px.

DEX treatment increased plasma MDA concentrations (*P* < .05, Figure 1(A)) and T-SOD activities (*P* < .05), whereas the activities of CAT (*P* = .069) and GSH-Px (*P* = .065) tended to increase under DEX treatment (Figure 1(B–D)).

**Figure 2.**
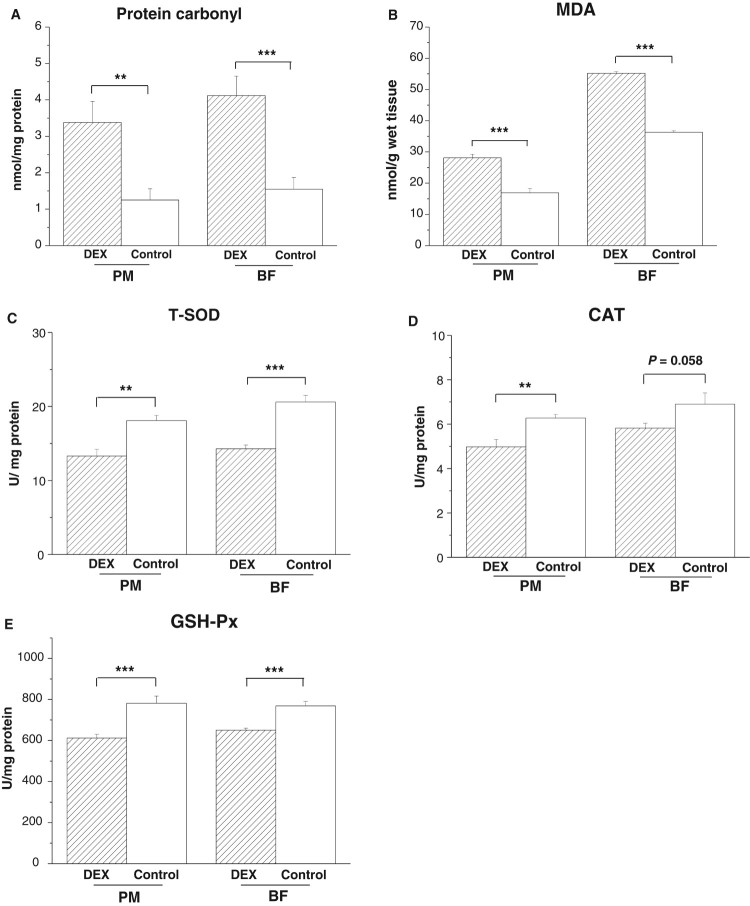
Effects of DEX (2.0 mg/kg body weight) on oxidative stress markers and antioxidant enzyme activities in the skeletal muscles (*M. pectoralis major*, PM; *M. biceps femoris*, BF) of the broilers in experiment 1. Data are expressed as the mean ± SEM (*n* = 8). **P < .01; ***P < .001. (A) Protein carbonyl; (B) MDA; (C) T-SOD; (D) CAT; (E) GSH-Px.

In both PM and BF, DEX treatment increased levels of protein carbonyl (*P*_PM_ < .01, *P*_BF_ = .001) and MDA (*P*_PM_ < .001, *P*_BF_ < .001) and decreased activities of T-SOD (*P*_PM_ < .001, *P*_BF_ < .001) and GSH-Px (*P*_PM_ < .001, *P*_BF_ < .001, Figure 2(A,B,C,E)). By contrast, CAT activity decreased under DEX in the PM (*P* < .01) and tended to decrease in the BF (*P* = .058, Figure 2(D)).

### Oxidative damage biomarkers and enzyme activities in mitochondria in experiment 1

**Figure 3.**
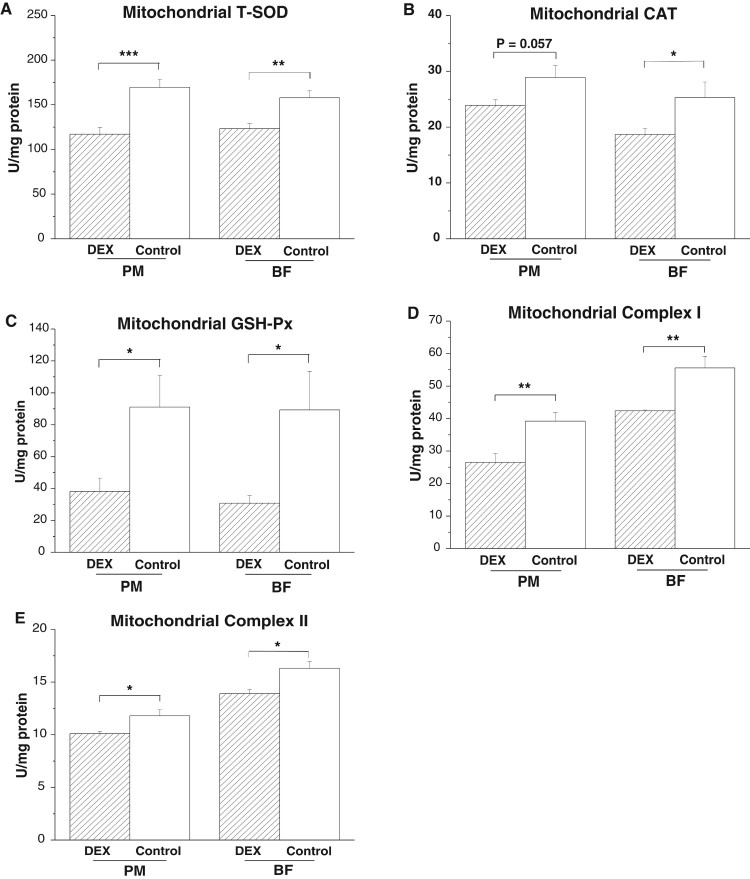
Effects of DEX (2.0 mg/kg body weight) on antioxidative enzyme activities and respiratory chain complex activities in the skeletal muscle mitochondria (*M. pectoralis major*, PM; *M. biceps femoris*, BF) of the broilers in experiment 1. Data are expressed as the mean ± SEM (*n* = 8). *P < .05; **P < .01; ***P < .001. (A) Mitochondrial T-SOD; (B) Mitochondrial CAT; (C) Mitochondrial GSH-Px; (D) Mitochondrial Complex I; (E) Mitochondrial Complex II.

DEX treatment decreased activities of T-SOD (*P*_PM_ < .001, *P*_BF_ < .01) and GSH-Px (*P*_PM_ < .05, *P*_BF_ < .05) in the PM and BF (Figure 3(A,C)). However, CAT activity decreased in the BF (*P* < .05) and tended to decrease in the PM (*P* = .057, Figure 3(B)) under DEX treatment. DEX treatment decreased the activities of complexes I (*P* < .01) and II (*P* < .05) in both PM and BF (Figure 3(D,E)).

### Oxidative damage biomarkers and enzyme activities in plasma in experiment 2

**Figure 4.**
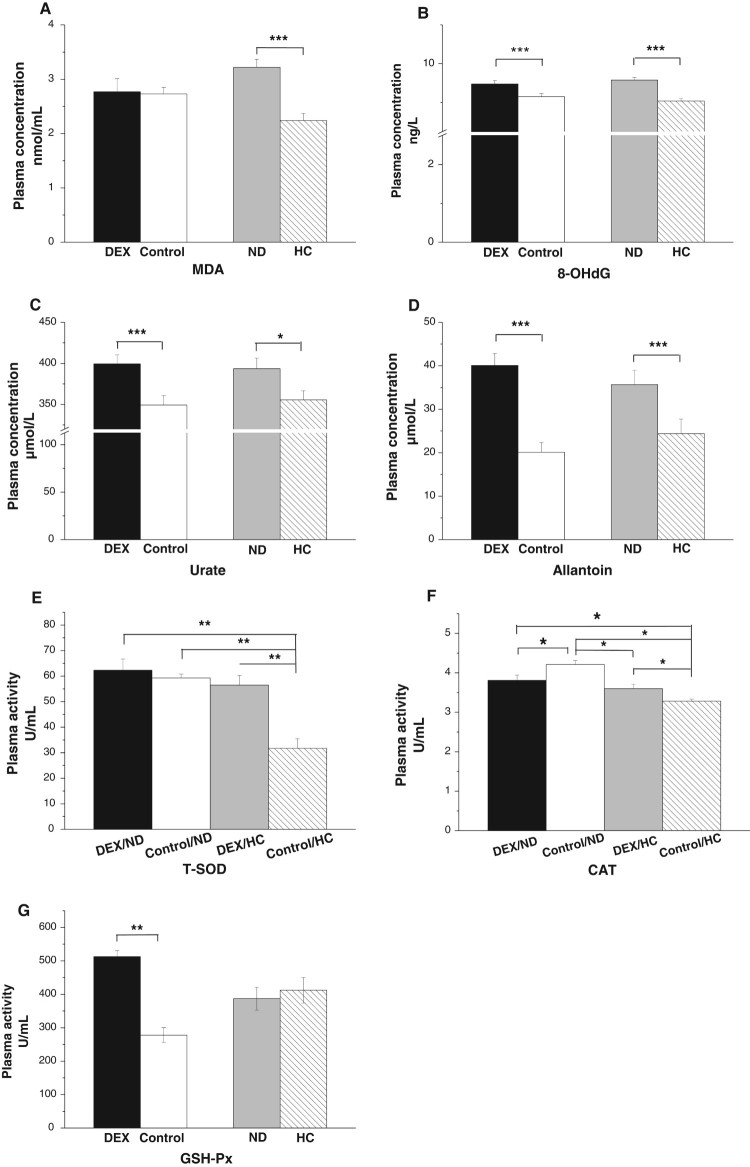
Effects of DEX (2.0 mg/kg body weight) and a HC diet rich in soy oil on the plasma concentrations of oxidative stress markers and activities of antioxidative enzymes of the broilers in experiment 2. Data are expressed as the mean ± SEM (*n* = 8). *P < .05; **P < .01; ***P < .001. (A) MDA; (B) 8-OHdG; (C) Urate; (D) Allantoin; (E) T-SOD; (F) CAT; (G) GSH-Px.

The interaction between the effects of DEX and diet treatments on plasma MDA, 8-OHdG, urate, and allantoin levels was not significant (*P* > .05). DEX treatment increased plasma concentrations of 8-OHdG (*P* < .001), urate (*P* < .001), and allantoin (*P* < .001, Figure 4(B–D)), whereas MDA levels were not significantly affected (*P* > .05, Figure 4(A)). By contrast, chickens fed the HC diet had lower levels of MDA (*P* < .001), 8-OHdG (*P* < .001), urate (*P* < .05), and allantoin (*P* < .001) than those of the ND-chickens (Figure 4(A–D)). The interaction between the effects of the DEX and diet treatments on CAT (*P* < .01) and T-SOD (*P* < .01) activities was significant (Figure 4(E,F)). DEX decreased T-SOD activity in HC-chickens (*P* < .01) but not in ND-chickens (*P* > .05; Figure 4(E)). By contrast, DEX decreased CAT activity in ND-chickens but increased CAT activity in HC-chickens (Figure 4(F)). GSH-Px activity increased (*P* < .001) under DEX treatment but was not altered by the different diets (Figure 4(G)).

### Oxidative damage biomarkers and enzyme activities in PM muscles and their mitochondria in experiment 2

**Figure 5.**
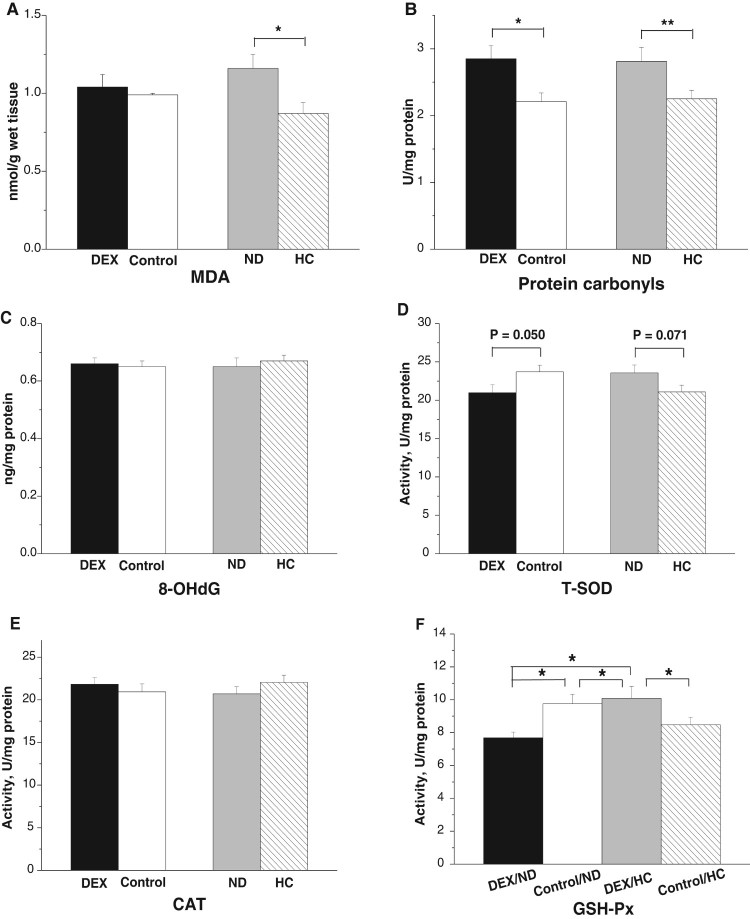
Effects of DEX (2.0 mg/kg body weight) and a HC diet rich in soy oil on the contents of oxidative stress markers and antioxidative enzymes in the *M. pectoralis major* (PM) muscle of the broilers in experiment 2. Data are expressed as the mean ± SEM (*n* = 8). *P < .05; **P < .01. (A) MDA; (B) Protein carbonyls; (C) 8-OHdG; (D) T-SOD; (E) CAT; (F) GSH-Px.

In the PM, the interaction between the effects of DEX and diet treatments was not significant for levels of MDA, 8-OHdG, and protein carbonyl (*P* > .05). DEX increased protein carbonyl levels (*P* < .05, Figure 5(B)) and decreased T-SOD activity (*P* = .05, Figure 5(D)) but had no influence on levels of MDA and 8-OHdG or on CAT activity (*P* > .05, Figure 5(A,C,E)). The interaction between DEX and diet on GSH-Px activity was significant (*P* < .01). DEX decreased GSH-Px activity in ND-chickens but activity increased in HC-chickens (Figure 5(F)). The HC diet decreased MDA (*P* < .05) and protein carbonyl levels (*P* < .01) and tended to decrease T-SOD activity (*P* = .071, Figure 5(A,B,D)). However, the different diet treatments had no effect on 8-OHdG levels (*P* > .05) or CAT activity (*P* > .05, Figure 5(C,E)).

**Figure 6.**
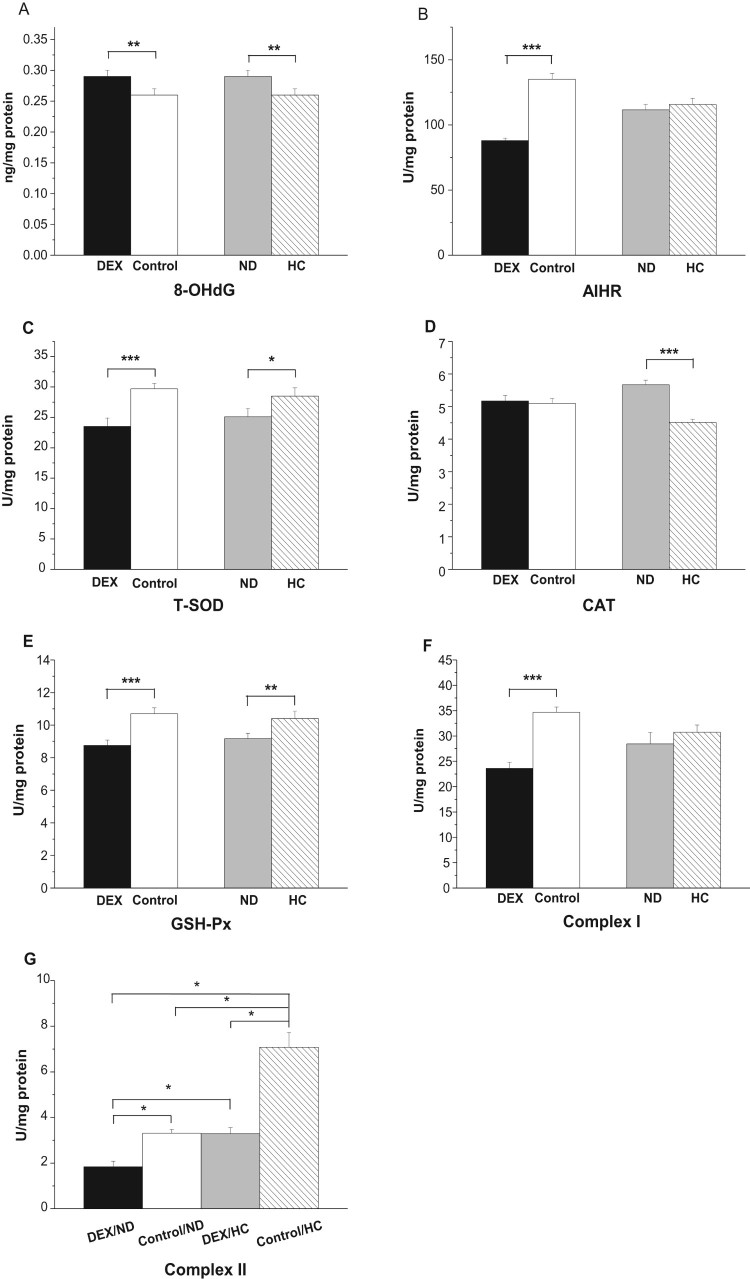
Effect of DEX (2.0 mg/kg body weight) and a HC diet rich in soy oil on the redox balance and respiratory chain complex activities in the mitochondria of the *M. pectoralis major* (PM) muscle of the broilers in experiment 2. Data are expressed as the mean ± SEM (*n* = 8). *P < .05; **P < .01; ***P < .001. (A) 8-OHdG; (B) AIHR; (C) T-SOD; (D) CAT; (E) GSH-Px; (F) Complex I; (G) Complex II.

In the mitochondria of the PM, the concentration of 8-OHdG increased (*P* < .01), whereas the AIHR decreased under DEX treatment (*P* < .001, Figure 6(A,B)). DEX treatment suppressed the activities of T-SOD (*P* < .001) and GSH-Px (*P* < .001) but not that of CAT (*P* > .05, Figure 6(C–E)). HC-chickens had significantly reduced levels of 8-OHdG (*P* < .01, Figure 6(A)). By contrast, the different diets had no significant effect on the AIHR (*P* > .05, Figure 6(B)). Diet significantly influenced T-SOD, CAT, and GSH-Px activities. Compared with ND-chickens, HC-chickens had higher activities of T-SOD (*P* < .05) and GSH-Px (*P* < .05), and lower activity of CAT (*P* < .001, Figure 6(C–E)).

The activities of complex I decreased under DEX (*P* < .001, Figure 6(F)), whereas DEX had a significant interaction with diet on complex II (*P* < .05): the activities of complex II were suppressed more severely in HC-chickens (53.2%) than in ND-chickens (44.1%, Figure 6(G)).

### Oxidative damage biomarkers and enzyme activities in BF muscles and their mitochondria in experiment 2

**Figure 7.**
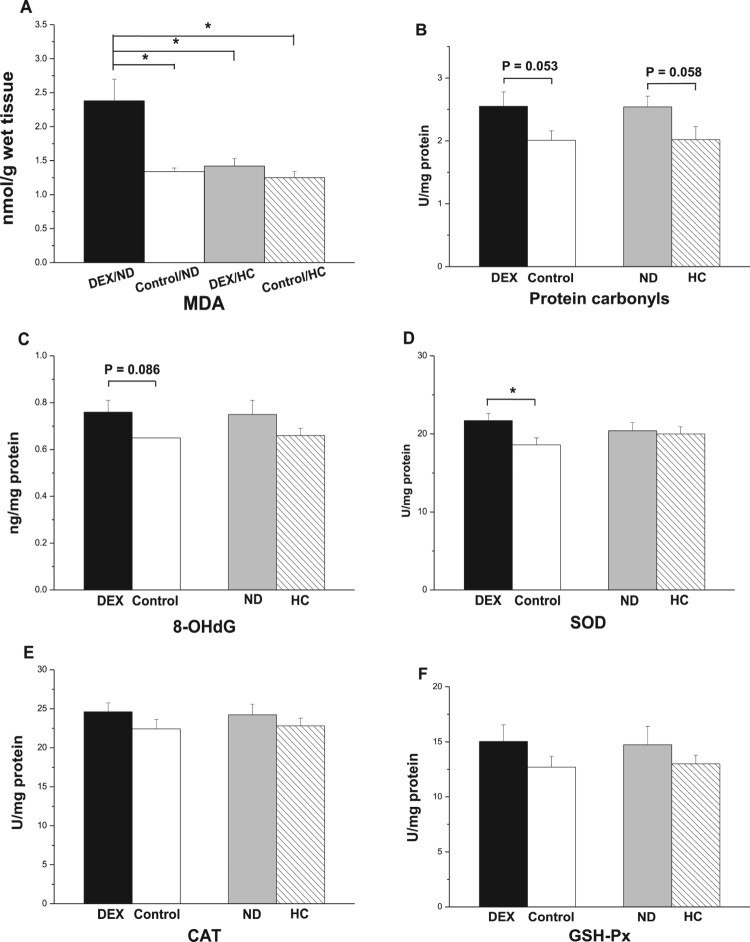
Effect of DEX (2.0 mg/kg body weight) and a HC diet rich in soy oil on the contents of oxidative stress markers and antioxidative enzymes in the *M. biceps femoris* (BF) muscle of the broilers in experiment 2. Data are expressed as the mean ± SEM (*n* = 8). *P < .05. (A) MDA; (B) Protein carbonyls; (C) 8-OHdG; (D) SOD; (E) CAT; (F) GSH-Px.

In the BF, the interaction between DEX and diet treatments had a significant effect on MDA levels (*P* < .01): DEX increased MDA levels in ND-chickens but not in HC-chickens (*P* < .01, Figure 7(A)). DEX treatment tended to increase levels of protein carbonyl (*P* = .053) and 8-OHdG (*P* = .086, Figure 7(B,C)). DEX treatment increased T-SOD activity (*P* < .05, Figure 6(D)), whereas CAT and GSH-Px activities were not affected (*P* > .05, Figure 7(E,F)). The HC diet tended to decrease protein carbonyl levels (*P* = .058), whereas 8-OHdG concentrations were not affected (*P* > .05, Figure 7(B,C)). Diet had no significant effect on the activities of T-SOD, CAT, and GSH-Px (*P* > .05, Figure 7(D–F)).

**Figure 8.**
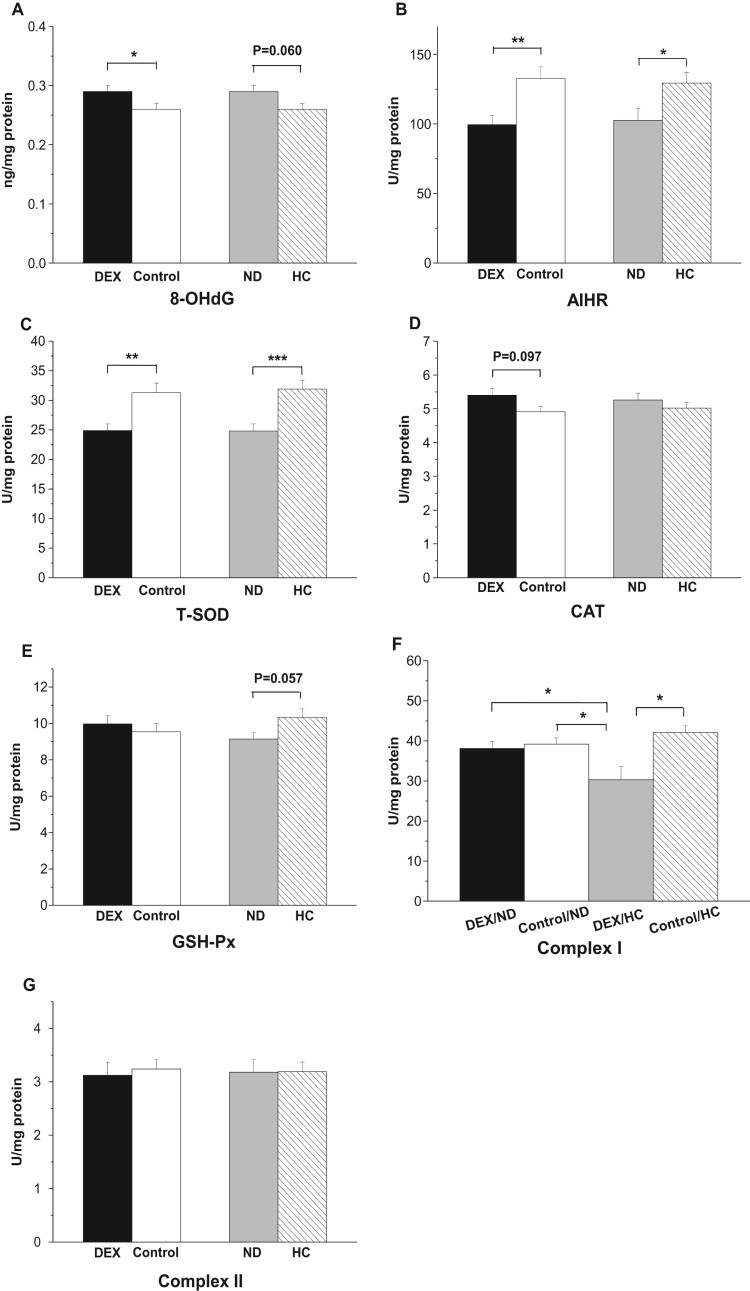
Effect of DEX (2.0 mg/kg body weight) and a HC diet rich in soy oil on the redox balance and respiratory chain complex activities in the *M. biceps femoris* (BF) muscle of the broilers in experiment 2. Data are expressed as the mean ± SEM (*n* = 8). *P < .05; **P < .01; ***P < .001. (A) 8-OHdG; (B) AIHR; (C) T-SOD; (D) CAT; (E) GSH-Px; (F) Complex I; (G) Complex II.

In the mitochondria of the BF, DEX treatment increased 8-OHdG levels (*P* < .05) but decreased the AIHR (*P* < .01, Figure 8(A,B)). DEX decreased mitochondrial T-SOD activity (*P* < .01) but had no significant effect on either GSH-Px or CAT activity (*P* > .05, Figure 8(C–E)). Compared with the results in the control groups, the HC diet increased the AIHR (*P* < .05) and tended to decrease 8-OHdG concentrations (*P* = .060, Figure 8(A,B)). HC treatment increased T-SOD activities (*P* < .001) and tended to increase GSH-Px activities (*P* = .057, Figure 8(C,E)). By contrast, CAT activities were not affected by the different diets (*P* > .05, Figure 8(D)).

The interaction between DEX and diet treatments was significant on the activities of complex I (*P* < .05, Figure 8(F)): the suppressive effect of DEX on complex I activity was only detected in HC-chickens (*P* < .01) and not in ND-chickens (*P* > .05). Neither the DEX nor diet treatment had any effect on complex II activity (*P* > .05, Figure 8(G)).

### Muscle fatty acid composition in experiment 2

**Figure 9.**
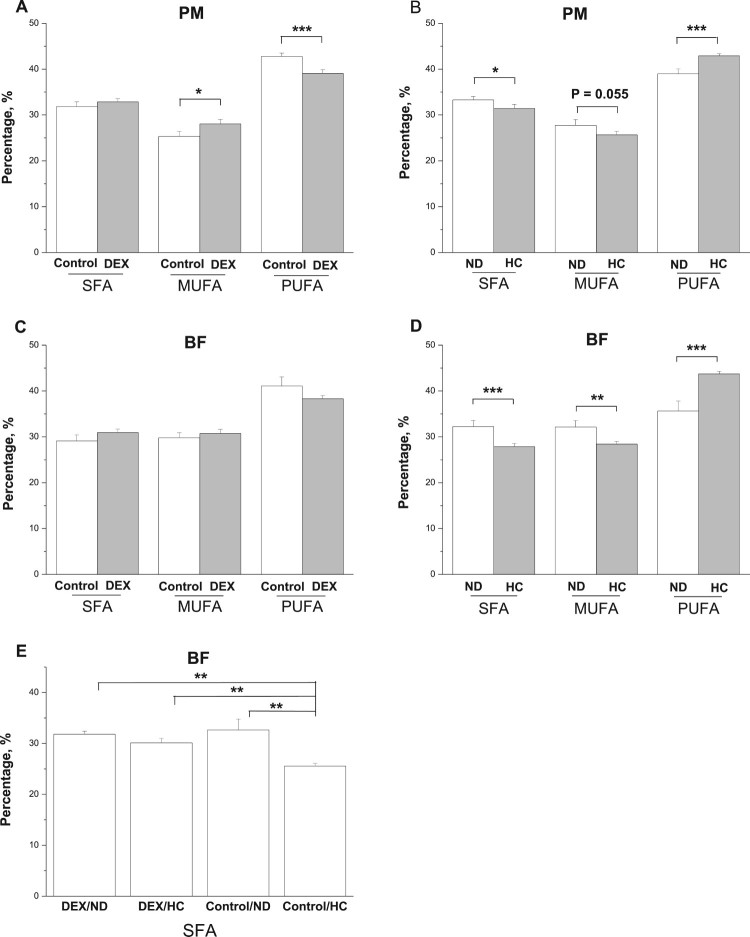
Effect of DEX (2.0 mg/kg body weight) and a HC diet rich in soy oil on the fatty acid composition of the skeletal muscles of broilers. Data are expressed as the mean ± SEM (*n* = 8). *P < .05; **P < .01; ***P < .001. (A) effect of DEX in PM; (B) effect of HC diet in PM; (C) effect of DEX in BF; (D) effect of HC diet in BF; (E) interaction effect of DEX and HC diet in SFA of BF.

In the PM, DEX treatment significantly decreased levels of PUFA (*P* < .001), increased those of MUFA (*P* < .05), and had no effect on SFA levels (*P* > .05, Figure 9(A)). The HC diet decreased levels of SFA (*P* < .05) and MUFA (*P* = .055) and increased those of PUFA (*P* < .001, Figure 9(B)). No interaction between the DEX and diet treatments on fatty acid composition was detected (*P* > .05).

In the BF, DEX had no significant effect on levels of SFA, MUFA, and PUFA (*P* > .05, Figure 9(C)). By contrast, the HC diet decreased (*P* < .01) levels of SFA and MUFA and increased those of PUFA (*P* < .001, Figure 9(D)). An interactive effect of the DEX and diet treatments was detected on SFA contents: the decrease in SFA levels caused by the HC diet was not detected in DEX-chickens (Figure 9(E)).

## Discussion

### DEX induced oxidative damage in plasma

DEX is an exogenous synthetic GC that is widely used to induce stress in animals. In a previous study, chickens treated with DEX for both short and long periods all showed elevated circulating corticosterone, indicating a stress response [31–34]. Oxidative stress is a major pathological mechanism involved in the maladaptation to chronic stress [35]. In this study, consistent with the results of previous studies [5–8], the GC induced oxidative damage in the plasma as demonstrated by the increased levels of plasma MDA in experiment 1 and the increased levels of 8-OHdG in experiment 2. This increase in oxidative damage might be caused by an increase in leakage of ROS, a diminished antioxidant capacity, or both. We evaluated non-enzymatic and enzymatic antioxidative systems. The increase in T-SOD (178.9%, *P* < .05), CAT (*P* = .069), and GSH-Px (*P* = .065) activities shown in experiment 1 in the DEX-chickens indicated that DEX treatment stimulated the antioxidant enzyme system. This conclusion was supported by the results of experiment 2 in which T-SOD (130.5%) and GSH-Px (184.8%) activities were elevated by the DEX treatment. The activity of CAT was unaltered, which might be related to the higher Km of CAT than that of GSH-Px [36,37].

Urate can serve as an antioxidant at normal physiological levels, and with an increase in plasma urate concentrations, non-enzymatic antioxidant capacities increase in GC-challenged chickens [6,7] and in healthy subjects suffering oxidative stress due to acute physical exercise [38]. In DEX-chickens, urate concentration (+14.4%) increased simultaneously with allantoin concentration (+103.3%), which is the end product of urate oxidation, indicating that urate was involved in the DEX-induced oxidative response [15]. However, urate can serve not only as an antioxidant but also as a pro-oxidant [39,40]. Therefore, the elevated allantoin levels might reflect peroxidase activity rather than the nonspecific scavenging of oxidants by urate [41]. Hence, the protective effect of urate against oxidative stress induced by DEX should be evaluated with caution.

### DEX induced oxidative damage in skeletal muscles is tissue specific

The two general types of skeletal muscle fibers are slow-twitch (type I) and fast-twitch (type II). Fast-twitch muscle fibers are thicker, quicker to contract, and wear out more rapidly than slow-twitch. For fast-twitch muscles, glycolysis is primarily used to produce usable energy, whereas slow-twitch muscles primarily use oxidative phosphorylation, which is slower but more efficient.

Consistent with the results of previous studies [5,8,35], based on the increase in levels of MDA and protein carbonyl in both PM and BF in experiment 1, DEX induced oxidative damage to lipids and proteins after 3 days of treatment. However, in experiment 2, in the PM, levels of protein carbonyl increased but not those of MDA and 8-OHdG, whereas in the BF, MDA levels increased, with a tendency for increase in protein carbonyl (*P* = .053) and 8-OHdG (*P* = .086) levels. These results indicated that DEX-induced oxidative damage depended on the muscle type, and that the slow-twitch muscle (BF) was more susceptible to oxidative stress induced by the GC. In the PM, the decrease in T-SOD and GSH-Px activities found in the two experiments and the suppressed CAT activity found in experiment 1 indicated that DEX impaired the antioxidant enzyme system in skeletal muscles, which might explain the oxidative damage induced by DEX in muscles. However, in the BF, the reduction in T-SOD, CAT, and GSH-Px activities observed in experiment 1 was not detected in experiment 2, and T-SOD activity was even elevated under DEX treatment. In rats, the expression of oxidative metabolism-related genes in slow-twitch muscles and fast-twitch muscles and their sensitivity to lipid peroxidation are different when provided with a diet supplemented with PUFAs [42]. Therefore, based on the results, DEX-induced oxidative stress in skeletal muscles occurred in a fiber-type-dependent manner.

Notably, the antioxidant enzyme activities in plasma varied in opposition to those in skeletal muscle. According to previous studies [5,8–10,43,44], we inferred that the elevated plasma antioxidant enzymatic activities indicated the oxidative damage of multiple organs, whereas the decrease in antioxidant enzymatic activities in skeletal muscles reflected the localized oxidative damage.

In the present study, the oxidative stress induced by DEX changed the fatty acid profile of skeletal muscles, particularly that of PM muscles. The increases in MUFAs and decreases in PUFAs in the PM were consistent with the suppressed activities of antioxidant enzymes. This result is also consistent with the work by Gao et al. [8] who reported that exogenous GC administration simultaneously induced oxidative damage and increased the saturation level of skeletal muscle fatty acids. Compared with mammals, the heart mitochondria of birds intrinsically have lower fatty acid double bond content [45]. The unsaturated degree of fatty acids in biomembranes is associated with their sensitivity to lipid peroxidation [46]. The low degree of fatty acid unsaturation in biomembranes of birds may confer advantage by decreasing their sensitivity to lipid peroxidation and preventing lipoxidation-derived damage to other macromolecules [47]. Hence, the results suggested that the GC-induced oxidative damage altered the fatty acid profiles by increasing the fatty acid saturation even in the skeletal muscles of chickens, which have an intrinsically higher degree of fatty acid saturation.

### Mitochondrial dysfunction is associated with increased oxidative damage

The mitochondrial respiratory chain is the primary site of ROS formation [12], and acute heat stress increases levels of ROS in the mitochondria of skeletal muscles [48]. Additionally, depressed activity of the mitochondrial respiratory chain is involved in the augmented production of ROS [49]. However, the relationship between mitochondrial function and oxidative stress in muscles induced by stress hormones remains to be elucidated.

The elevated 8-OHdG levels found in the PM and BF muscles indicated an increase in mitochondrial DNA damage as a result of the DEX treatment. In vitro, DEX causes DNA damage via oxidative stress [50]. In humans, long-term corticosteroid administration increases 8-OHdG concentrations in association with mitochondrial DNA damage in skeletal muscles [51]. In our previous study, heat exposure-induced oxidative stress caused an increase in mitochondrial 8-OHdG in the breast and thigh muscles [15]. These results suggest that DEX induced oxidative damage in skeletal muscle mitochondria. Based on the decrease in the AIHR, which was used to assess antioxidant capacity for reducing hydrogen peroxide [52], and the suppression of mitochondrial T-SOD activity, the capacity of mitochondria of skeletal muscles for reducing ROS and hydrogen peroxide was impaired. In humans, hydroxyl radicals play a role in the pathogenesis of steroid myopathy [53]. In thymocytes, DEX administration significantly decreases antioxidant enzyme activities and significantly increases ROS production, lipid peroxidation, mitochondrial dysfunction, caspase-3 activation, and cellular apoptosis [54]. Therefore, these results suggest that a suppressed scavenging capacity played an important role in DEX-induced mitochondrial oxidative damage in skeletal muscles.

The oxidative phosphorylation system consists of five multiprotein complexes, complexes I to IV and ATP synthase (complex V), through which electrons from electron donors pass to oxygen. Electrons may leak from the respiratory chain when moving along the transport chain [12]. The principal site of superoxide generation in mitochondria is complex I [55,56]. In mammals, inhibition of complex I results in greater ROS production rates and increased oxidative stress [13,14]. Our previous study found that a reduction in the activity of complex I rather than that of complex III was primarily responsible for the increased leakage of superoxides in the breast and thigh muscles of heat-stressed broilers [15]. In this study, complex I activity was suppressed by DEX in both PM and BF, suggesting that impaired complex I activity was involved in DEX-induced mitochondrial oxidative stress. In rat muscle mitochondria, the quinol site in complex I and the flavin site in complex II generate approximately half of the hydrogen peroxide produced [57]. However, the suppression of mitochondrial complex II activity in the PM and not in the BF implied that complex I was the primary site involved in DEX-induced ROS production. This result is consistent with that of Bottje et al. [29] who reported that complex I might be a potential site of electron leakage in the muscle. In the present study, the activities of complex III and IV were not determined, and therefore, their role in the leakage of electrons could not be excluded; none of the mitochondrial parameters were normalized with the activity of citrate synthase or other mitochondrial proteins. Hence, the results herein should be interpreted with caution.

### A HC diet rich in soy oil alleviated oxidative stress

In humans, lipotoxicity is an important factor of metabolic diseases, and the oxidative stress induced by a high-fat diet is involved in the development of these diseases. High fat diet-induced obesity is accompanied by increased oxidative stress in hepatic, cardiac, and renal tissues. This stress is characterized by a reduction in antioxidant enzyme activities and glutathione levels, which correlates with increases in MDA and protein carbonyl levels [58]. In the present study, the chickens fed a HC diet rich in soy oil showed decreased plasma concentrations of all measured oxidative biomarkers, including MDA and 8-OHdG (*P* < .001), indicating a decline in oxidative damage. By contrast, the reduction in T-SOD and CAT activities, in addition to the elevated AIHR, implied that the antioxidative system was not stimulated in HC-chickens. Providing support for this conclusion were the reductions in concentrations of oxidative damage markers, such as MDA and protein carbonyl, in the PM and BF of HC-chickens, with the activities of T-SOD, CAT, and GSH-Px not significantly altered. Consistent with our results, bilateral ovariectomized rats fed a standard diet with 15% soy oil showed an improved antioxidant status [59]. However, long-term (4 months) and higher supplemental levels (20%) of soy oil may increase the levels of lipid peroxidation products because of the high percentage of PUFAs [60]. In this study, a HC diet rich in soy oil changed the fatty acid profiles of both PM and BF muscles: PUFAs increased but SFAs and MUFAs decreased, consistent with previous reports [46,61]. Hence, the results suggested that chickens fed a HC diet rich in soy oil over a relatively short period suffered less oxidative damage.

In skeletal muscles, oxidative stress is a negative regulator of the actions of insulin following a high-fat feeding. A high-fat diet results in the downregulation of the genes necessary for oxidative phosphorylation and mitochondrial biogenesis, which may result in mitochondrial dysfunction [62]. The inhibition of oxidative phosphorylation increases mitochondrial ROS production, eliciting antioxidant defenses and resulting in an increased mtDNA mutation rate [63]. A short-term (28-day) high-fat feeding (45% energy from palm oil) treatment increased the saturation of phospholipids in the mitochondrial membrane in the skeletal muscles of rats [64]. By contrast, dietary fat in the form of plant oils rich in PUFAs can benefit the redox balance [23,24,65]. In the present study, the decreased levels of 8-OHdG in the mitochondria from the PM and BF muscles of HC-chickens indicated that a relatively lower level of oxidative damage occurred in the HC-chickens than that in the ND-chickens. The increase in T-SOD and GSH-Px activities associated with the HC treatment was likely responsible for the reduced level of oxidative damage in the mitochondria of the HC-chickens. The lack of an effect on activities of complex I and II, except for the elevation in complex II activity in the PM, indicated that mitochondrial function was not impaired by the HC rich in soy oil diet used in the present study. This result suggested that moderate levels of dietary fat in the form of soy oil were beneficial for the maintenance of redox balance in skeletal muscles of chickens.

### A HC diet rich in soy oil alleviated the oxidative stress induced by DEX

In the present study, the interaction between HC diet and DEX treatment was further investigated. Although a significant increase in MDA levels was observed in the DEX-ND chickens, no significant interactive effect of DEX and diet was observed on the levels of all oxidative damage biomarkers measured in the plasma, skeletal muscle, and mitochondria. These results indicated that the oxidative damage induced by DEX could be alleviated by a HC diet rich in soy oil. By contrast, the interactive effect of DEX and diet on complex II in the PM (HC, −53.2% vs. ND, −44.1%) and on complex I in the PM (HC, −28.0% vs. ND, −2.7%) indicated that the DEX-induced suppression was more severe in the HC-chickens. In rats subjected to 2 weeks of caloric restriction and then fed a high-fat diet (430 kJ ME/day) for 1 week, mitochondrial efficiency and oxidative damage increased in their skeletal muscles [22]. Hence, these results implied that the HC diet increased the production of ROS during a DEX challenge. In HC-chickens, DEX increased the activities of T-SOD and CAT in plasma and GSH-Px activity in the PM, which were unchanged or suppressed by DEX in the ND-chickens, indicating an alteration in the antioxidant capacity. In rats that were fed for 1 week an isocaloric high-fat diet, the antioxidant defense systems increased in their skeletal muscles [20]. A moderate oxidative stress can induce cellular antioxidant response in vascular cells and therefore could be beneficial for preventing further oxidative stress [66]. Collectively, these results suggested that a HC diet rich in soy oil stimulated antioxidant defenses in DEX-chickens.

**Figure 10.**
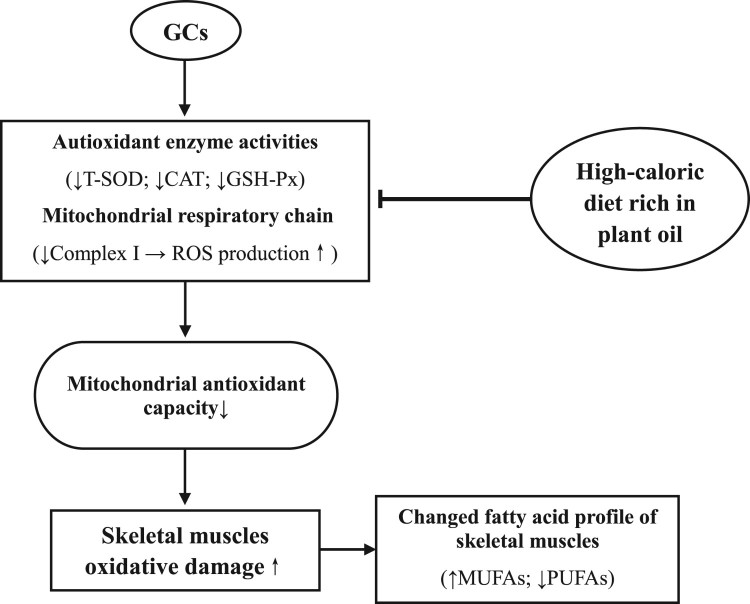
Proposed model of GCs and soy oil diet action on oxidative damage of skeletal muscles in chickens (↑ increase; ↓ decrease; ⊥ inhibitory). GCs suppresses the mitochondrial complex I activity, decreases the unsaturated degree of fatty acid profiles, and induces oxidative damage in skeletal muscles. A HC diet rich in soy oil improves antioxidative capacities and reduces the oxidative stress induced by the GCs.

In conclusion, DEX induced oxidative damage in the plasma, skeletal muscles, and muscle cell mitochondria of chickens and decreased the unsaturated degree of fatty acid profiles in skeletal muscles. The suppressed activity of mitochondrial complex I was involved in the oxidative damage in skeletal muscles and mitochondria. The HC diet rich in plant oil improved antioxidative capacities and reduced the oxidative stress induced by the GCs (Figure 10). A mechanistic study investigating the activation of Nrf2 and the expression of genes related to antioxidant enzymes should be conducted in the future.
